# Advances in Photopletysmography Signal Analysis for Biomedical Applications

**DOI:** 10.3390/s18061894

**Published:** 2018-06-09

**Authors:** Jermana L. Moraes, Matheus X. Rocha, Glauber G. Vasconcelos, José E. Vasconcelos Filho, Victor Hugo C. de Albuquerque, Auzuir R. Alexandria

**Affiliations:** 1 Programa de Pós-Graduação em Engenharia de Telecomunicações, Instituto Federal de Educação, Ciência e Tecnologia do Ceará, Fortaleza 60040-531, Ceará, Brazil; jermanalopes@gmail.com (J.L.M.); matheusxavier225@gmail.com (M.X.R.); auzuir@ifce.edu.br (A.R.A.); 2 Hospital de Messejana–Dr. Carlos Alberto Studart–Avenida Frei Cirilo, 3480–Messejana, Fortaleza 60846-190, Ceará, Brazil; glaubergeanv@gmail.com; 3 Programa de Pós-Graduação em Informática Aplicada, Laboratório de Bioinformática, Universidade de Fortaleza, Fortaleza 60811-905, Ceará, Brazil; euricovasconcelos@unifor.br

**Keywords:** heart rate variability, photoplethysmography, cardiovascular diseases, Internet of Health Things, health care

## Abstract

Heart Rate Variability (HRV) is an important tool for the analysis of a patient’s physiological conditions, as well a method aiding the diagnosis of cardiopathies. Photoplethysmography (PPG) is an optical technique applied in the monitoring of the HRV and its adoption has been growing significantly, compared to the most commonly used method in medicine, Electrocardiography (ECG). In this survey, definitions of these technique are presented, the different types of sensors used are explained, and the methods for the study and analysis of the PPG signal (linear and nonlinear methods) are described. Moreover, the progress, and the clinical and practical applicability of the PPG technique in the diagnosis of cardiovascular diseases are evaluated. In addition, the latest technologies utilized in the development of new tools for medical diagnosis are presented, such as Internet of Things, Internet of Health Things, genetic algorithms, artificial intelligence and biosensors which result in personalized advances in e-health and health care. After the study of these technologies, it can be noted that PPG associated with them is an important tool for the diagnosis of some diseases, due to its simplicity, its cost–benefit ratio, the easiness of signals acquisition, and especially because it is a non-invasive technique.

## 1. Introduction

One of the fundamental characteristics of the heart is to be able to change its heartbeat rate. These spontaneous fluctuations of the heart rate (HR) reflect the relationship between ongoing interferences in the cardiovascular system and the response of its regulatory mechanisms [1].

One of the methods used to evaluate cardiovascular autonomic nervous system activity is HRV analysis (cranial heart rate variability) [2]. The autonomic nervous system is responsible for the connection of the central nervous system to the cardiovascular system. The heart rate variability is constantly modulated through complex interactions between branches of the autonomic nervous system, the sympathetic nervous system, and the vagus nerve [3]. Since the activity of the autonomic nervous system and the heart rate are related in a nonlinear manner, the changes in the sympathetic activity or the vagal tone have the ability to change the response of the heart rate to the stimulation of any branch of the system [3,4].

Neural control is completely related to heart rate (HR) and baroreceptor activity [5]. Therefore, through a complex interaction of stimulus and blocking, the responses of the sympathetic and parasympathetic pathways that change the HR are developed, adapting it to the needs of each moment. The increase in HR is a consequence of the greater action of the sympathetic pathway and the lower parasympathetic activity. Therefore, the activities of the sympathetic pathway increase HR while the activities of the parasympathetic path reduce it [6,7]. With the discovery of the relationship between the autonomic nervous system and mortality from cardiovascular diseases, it has become necessary to study the increase in sympathetic activity and the reduction of parasympathetic activity, which are conditions found in several cardiovascular diseases [8].

**Figure 1:**
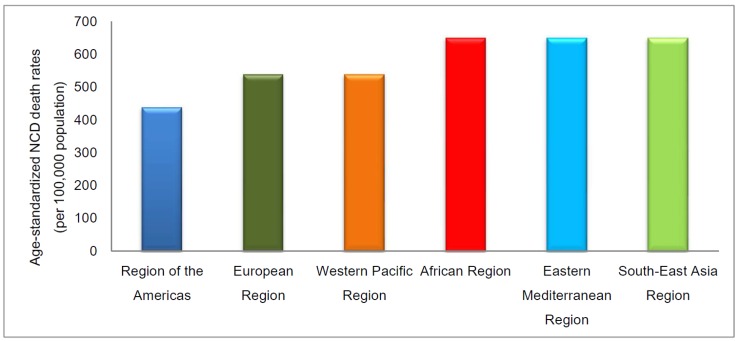
Mortality rates by NTCD per 100,000 habitants, all ages, for region of WHO, 2012 [9].

Heart diseases are considered a major public health problem, since they are the leading cause of death worldwide, especially in populations of large urban centers. According to data from the World Health Organization, 17.3 million people died in 2012 as victims of this type of disease. The estimate is that, by 2030, this number will be 23.6 million [9,10]. The statistical data of death by NTCDs separated by WHO region can be verified in Figure 1.

Mortality rates are steadily rising. Therefore, it is important to adopt preventive measures and control various risk factors, such as hypertension, diabetes, high cholesterol, smoking, alcoholism, stress, obesity and physical inactivity. Medical consultations are essential for continuous evaluation to reduce the number of deaths due to cardiovascular diseases [11,12].

The analysis of HRV signals is important when studying the autonomic nervous system, as it supports the evaluation of the balance between the sympathetic and parasympathetic influences in the cardiac rhythm [2]. The heart rate variability is a valuable vital signal, which reflects the physical condition of a patient [13]. A misshapen value between heartbeats is one of the first indicators of the existence of an anomaly in the patient’s health. It can reveal diverse conditions, such as respiratory and cardiac arrest, systemic inflammatory response syndrome, renal insufficiency, cardiac insufficiency, systolic arterial pressure, among others [14]. Among the methods used to determine the heart rate, photoplethysmography estimates the alterations in the blood flow adopting an optical method [15].

Photoplethysmography (PPG) is one on the most popular technologies in the last decade for monitoring of the physiological conditions of a patient, and, because it is an non-invasive method, PPG has been largely applied to personal portable devices and pulse oximetry due to its convenience and capacity to perform continuous readings. In addition, the signal can provide information about both the cardiovascular and respiratory systems. The vast viability of the utilization and easiness of the patient’s physiological data acquisition characterize this method [13,16]. Compared to the electrocardiogram (ECG) signal [17,18], the PPG signal does not have a complex hardware implementation. It also does not have the requirement of a reference signal, hence the PPG sensors can be incorporated into wristbands. The utilization of these systems then becomes more accessible than the current ECG monitoring systems, which require electrodes to be attached on the patient’s chest [19,20,21].

In the sense of adding elements to the literature referent to a non-invasive technique, of easy utilization, which presents valuable information about the cardiovascular system, it was understood as a suitable theme to be investigated. Thus, the presentation of current information referent to PPG, such as concepts, analysis methods, variations of the application methods of the technique, means of interpretation of results and clinical applicability, constitutes a contribution either to researchers of diverse fields, or to clinical professionals working in the wide range of health areas. Furthermore, it presents a database and information for hospitals and clinics.

While analyzing some of the literature about the theme of study, photoplethysmography, various papers emphasizing the importance of a more detailed and specific study about PPG were found. The work in [22] presents a review of the method of estimation of the heart rate using facial skin and digital cameras, highlighting the calculation of the HRV by PPG. Additionally, the work in [23] presents a review of the literature about estimation of the respiratory rate using ECG and PPG. In addition, in the work in [24], the objective of the researchers was to display the utilization of the PPG technique for clinic applications, comparing it to ECG and presenting the importance of the determination of the HRV and the Partial Thromboplastin Time (PTT).

The distinctive aspect of the present paper is in the elaboration of a complete review of the PPG technique, a comparison with the ECG method, and a description of the most important variables (HRV, PTT, Pulse Rate Variability (PRV), Pulse Wave Velocity (PWV), etc.) which can be extracted from the PPG signal and used in a thorough analysis of the physiological conditions of an individual.

This paper is structured as follows. In Section 1, an overview is presented on the global statistical data on cardiovascular diseases, some fundamental concepts of HRV and a brief introduction on the technique of photoplethysmography (PPG). Section 2 presents the details of the photoplethysmography technique (PPG), explaining its principle of operation and the types of PPG sensors, as well as the metrics obtained with this technique and how to improve those metrics. Section 3 explains the linear and nonlinear methods for PPG signal analysis, differentiating these methods in relation to the time and frequency domains. Section 4 explains some techniques for PPG sensor instrumentation and improvement techniques for PPG signal analysis. In Section 5, some important studies on the PPG technique are discussed, correlating this technique to the diagnosis of heart diseases, as well as the use of PPG in several technologies. In Section 6, a general discussion is presented on the themes mentioned in previous sections. In Section 7, the lessons learned about the studied subject are discussed. Finally, in Section 8, the conclusion of the study is presented.

## 2. Photoplethysmography

The sequence of events that happen between the beginning and the end of a heartbeat is called the cardiac cycle [25]. The cardiac cycle is composed of two main phases: the ventricular diastole and the ventricular systole. In the diastole, or relaxation phase, the blood flows to the auricles, causing pressure to decrease in the blood vessels. In the systole, or contraction phase, the blood is pumped out of the ventricles and distributed throughout the body, causing pressure to increase in the blood vessels [25,26].

Several methods and devices perform heart rate measurement and heart monitoring [27]. Electrocardiogram (ECG), analog converters and cardiofrequencimeters are the main equipment used to measure heart rate variability [8]. Similarly, photoplethysmography (PPG) is also used for measuring the HRV. It is considered a portable, low cost technology of simple utilization, also being non-invasive and applicable in diverse environments. Besides all previously mentioned attributes, the development of signal processing algorithms adds robustness, contributing to the evolution of this technology [28].

Photoplethysmography (PPG) is a non-invasive technique for measuring blood perfusion through tissues by the emission of light rays [29,30]. Researchers from around the world, beginning in 1939, have indicated the need for blood circulation studies using noninvasive techniques. Consequently, an electronic device was developed for measuring blood volume and blood flow, namely plethysmography [31]. The clinical fitness of the photoplethysmography was presented by Alrick Hertzman in 1937 when describing the use of a reflexive photoplethysmography system, measuring variations in the blood volume, induced by Valsalva maneuver, in the finger of patients [32].

**Figure 2:**
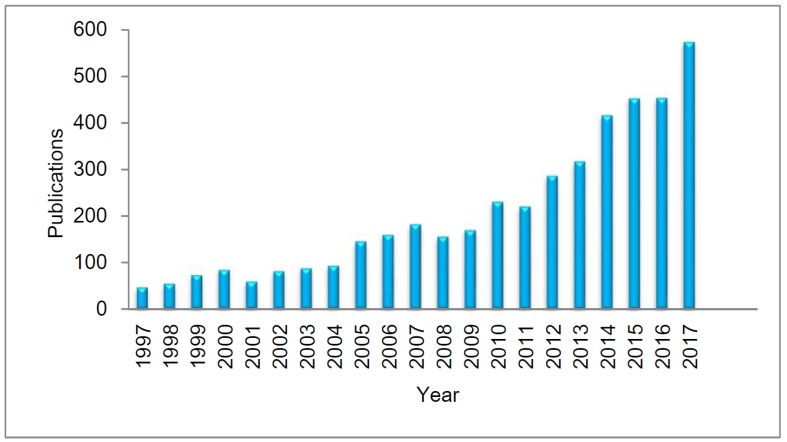
Comparative of 20 years (1997–2017) of PPG publications. Data were obtained from Web of Science ${}^{TM}$ using “photoplethysmography” as topic (accessed on 20 February 2018).

PPG is a simple and inexpensive tool that can be defined as an optical biomonitoring technique used to measure changes in blood volume in microvascular tissue under the skin occurring due to the blood pulsatile nature [25]. PPG signal extraction is considered simple; however, the components of this signal can provide valuable information about the cardiovascular system [25,33]. Over the past 20 years, there has been a significant increase in the number of papers published regarding the PPG technique, as shown in Figure 2. The popularity of this technique is due to the important applications in the evaluation of the cardiovascular system, signal monitoring and detection of oxygen in blood [25].

Photoplethysmography can measure the heart rate, that is, the alterations of blood flow, detecting changes in the blood volume [15]. A photo-emitter of infrared light is coupled to a photo-receiver, using as the medium of light propagation the body segment in which is desired to register the plethysmographic signal. The pulsatile signal of the blood volume (pulse wave) is detected by the photo-transistor as a modulation of the original signal of the carrier wave [34]. The indicated wavelength of the infrared photo-emitter is close to 940 nm [35]. However, according to the same author, this technique allows extraction of values of some physiological parameters of a patient, such as the variability of the time between heartbeats and, after processing of such parameters, the heart rate.

**Figure 3:**
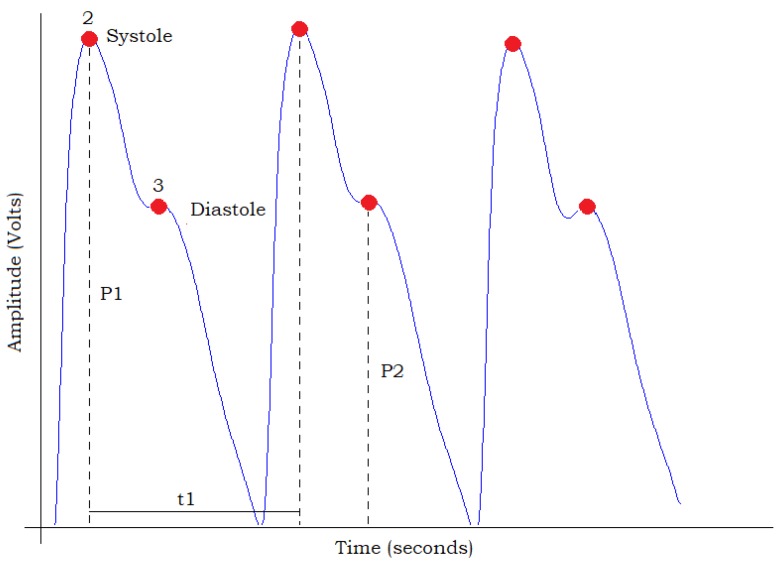
PPG signal analysis.

The photoplethysmographic wave describes changes in the attenuation of light energy in its pathway when transmitted or reflected in tissues and bloodstream. This waveform is totally related to the systole and the diastole of the cardiac cycle [36], as can be observed in Figure 3. The PPG signal represented in Figure 3 was extracted from a healthy person.

The waveform of the PPG signal describes the variations in the attenuation that light energy suffers on its path when transmitted or reflected in biological tissues [36]. Based on the analysis in Figure 3, it is possible to estimate some parameters, such as the amplitude of the systole pulse wave (P1), amplitude of the diastole pulse wave (P2), time interval between beats (t${}_{1}$), etc. From this, it is possible to determine the instant heart rate (HR${}_{inst}$) and the mean (HR${}_{med}$). The HR${}_{inst}$ can be calculated from the interval between beats (t${}_{1}$); using Equation (1), one can calculate the instantaneous HRV.

(1) $$HR_{inst}=\frac{60}{t_{1}}$$

(2) $$HR_{med}=\frac{1}{Q_{nn}}\sum_{k\in [T_{i},T_{f}]}NN\left[k\right]$$

where $Q_{nn}$ corresponds to the amount of samples of normal intervals (NN) in the interval [$T_{i}$, $T_{f}$] [37].

The increase in heart rate and pulse wave amplitude (P1), shown in Figure 3 with number 2, reflects the growth of blood flow in the signal due to contraction of the left ventricle of the heart. The amplitude of the dicrotic minimum, represented in Figure 3 with number 3, varies with arterial vascular elasticity and fundamentally depends on the interaction of the initial pressure wave, when the heart contracts, and with the pressure wave that is reflected due to peripheral arteries [15]. However, the points identified in Figure 3 may not be present in all PPG signals, since the waveform of the photoplethysmography signal changes significantly as a function of some conditions, such as body age, vascular age, physical status (regarding sleeping hours, physical activities, etc.) and others [15,28].

The most commonly used method for analysis of the PPG signal is to detect its peak values, corresponding approximately to the systolic phases of the cardiac cycle, and register the time passed between maximum PPG successive values, as explained before. Although the time of the peak in a PPG signal depends on many factors, including the arterial rigidity, arterial pressure, pulse wave velocity, and distance of the local of measurement of the aorta, among others. Thus, an alternative to the PPG peak method is to take the difference between “foot points” of consecutive PPG pulses [38].

To stipulate the foot points, it is necessary to study and calculate the following items [38]:

- Maximum first derivative: Equals the maximum positive pulse gradient, i.e., the maximum rate of upswing of the pulse wave signal corresponding to the peak velocity of the vessel wall. This is determined numerically from the maximum positive value of the first derivative of the pulse wave.
- Maximum second derivative: Equals the maximum positive rate of change of the gradient, i.e., the maximum acceleration of the vessel wall. This is determined from the calculation of the maximum positive value of the second derivative of the pulse wave.

There is no exact definition for the “standing point” position of a pulse wave, and there are many alternatives to determine them. These methods make use of the determination of the minimum value of the pulse wave signal, the maximum gradient of the first derivative or the second maximum derivative of the pulse signal. However, there is also a more complex approach which is called “tangent intersection” in which two preliminary points are determined using two different methods (for example, first maximum derivative and minimum value) and the intersection point of tangent lines to the waveform in each foot defines a third point [38,39,40].

There are more recent methods, such as the “diastolic patch” technique where the two foot point regions of two waveforms are correlated to find the time difference between the times of arrival of these waveforms [38,41]. Furthermore, some important variables can be estimated using the PPG signal; one of them is the PTT (Pulse Transit Time). PPT is defined as the time the pulse propagates from the heart to a peripheral locale and has been proposed as a potential substitute of the calculation of the arterial pressure (AP). That is, the time required for the arterial pulse pressure wave to propagate from the aortic valve to a peripheral site (usually the finger) is considered. The PTT may be promptly derived from the electrocardiogram (ECG) or photoplethysmogram (PPG) [42,43,44]. The rigidity and the tension in the arterial walls are the fundamental causes that determine the speed of transmission of the pulse wave, and this, in turn, depends to a great extent on the blood pressure [42,43].

**Figure 4:**
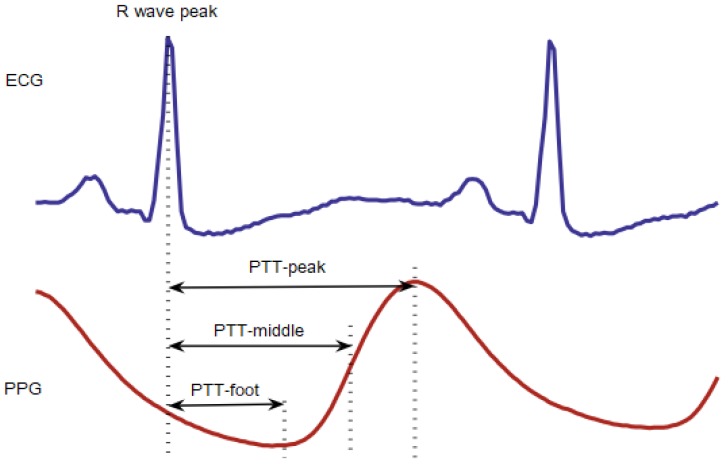
Different measurement points of PTT [45].

In the determination of the PTT from the ECG, it is considered the time interval between peak of the waveform R, and, for the photoplethysmogram, it is considered a characteristic point of the PPG in the same cardiac cycle. The three different points of measurement of the PTT are represented in Figure 4 and are defined as: PTT-peak, PTT-middle, and PTT-foot. PTT-middle is the maximum derivative point [45].

The PTT value is inversely proportional to the blood pressure (BP) value, so its evaluation is considered a promising method for continuous, noninvasive monitoring. The most commonly used technology for detecting the distal pulse waveform is photoplethysmography (PPG). However, such pulse transit time measurements are in fact a measure of pulse arrival time (PAT) rather than PTT, and are used as a substitute for PTT [46,47]. However, several studies have commented on the reliability of PTT with PAT evaluations, since it can be altered by variations of the pre-ejection period (PEP) [46]. The relationship between the PAT and the PTT can be analyzed with Equation (3).

(3) PAT=PET+PTT

As a solution to this problem, there is the cardiographic impedance (ICG) technology that has been applied for the detection of aortic valve opening as a reference time for proximal location. Although this method provides more accurate estimates than the use of ECG R peaks, ICG based systems are not widely used, as the signal quality is poor. Additionally, these systems are inconvenient due to the need for multiple electrodes around the body [46,47].

Clinically, the PTT is highly utilized in the investigation of diseases related to sleep, such as sleep apnea, being a tool frequently used in the identification of obstruction of the upper airways and the increase in the respiratory effort during sleep, since it causes a drop in the arterial pressure and stretching of the PTT. For this reason, the obstructive apneas are associated to the increase in amplitude of the PTT oscillations as indication of respiratory effort [43,48].

Another important variable to be calculated is the pulse wave velocity (PWV), which provides relevant information about the heart rate as well the good functioning of the heart. The PWV is associated with the calculation of the elasticity of the blood vessels and the arterial pressure values. These measurements are considered complex indicators for the state of the cardiovascular system [30,49,50].

The increase of the arterial rigidity is a complex phenomenon characterized by the decrease of the complacency (or distensiblity) of the great arteries. The phenomenon occurs with aging, as well as in presence of diseases associated with the cardiovascular system, such as arterial hypertension, diabetes, dyslipidemia and obesity. These diseases are pointed out as potential promoters of the increase of arterial rigidity [51]. Clinically, the increased arterial rigidity can be manifested as consequence of the increase of pulse pressure (PP) and the isolated systolic hypertension, being the pulse wave velocity (PWV) considered a gold standard for evaluating the arterial rigidity. Thus, the increase in the pulse wave velocity is related to the increase of the arterial rigidity [51,52]. Equation (4), for the calculation of the PWV, is presented.

(4) $$PWV=\frac{\Delta D}{\Delta T}$$

where ΔD relates to the distance between heartbeats, while ΔT is related to the time between heartbeats.

Pulse Wave Velocity (PWV) is the displacement velocity of a pressure wave through an arterial segment and is commonly used as an early diagnostic variable for cardiovascular risk and an important marker in the role of primary prevention of arterial pathology. The greater is the PWO, the greater is the arterial rigidity as well as the underlying cardiovascular risk [53].

In the work in [54], a method was developed to estimate PWV using signals from circulatory waves derived from multiple PPG sensors. The method manipulates two wearable PPG in-line sensors placed at a distance known from one another in the ulnar and digital artery. The results showed that the method is able to measure changes in arterial PWV that result from fluctuations in mean arterial pressure. The PTT and consequently the VTP are influenced by elastic properties, mainly intrinsic, of the arterial wall, such as age, the vascular remodeling, arteriosclerosis, and blood pressure [42,43,48].

In addition to these variables, Pulse Rate Variability (PRV) can also be extracted from the PPG. It was studied as a potential substitute for the heart rate variability value. As the PPG also allows acquiring physiological parameters, such as blood oxygenation and the ventilatory rate, the use of PRV instead of HRV could be particularly suitable to these applications where the simultaneous acquisition of many signals is necessary, for example in studies of sleep disorders, especially for studies of ambulatory sleep. The calculation of the PRV is related to the PTT, that is, the beat-to-beat alterations in the pulse wave velocity [55].

Some studies report the possibility of using PRV as an alternative solution to HRV, and these studies were carried out under stationary conditions, using invariant analyses in time. However, in situations involving non-stationary processes and significant changes in the autonomic balance, such as the orthostatic test, Valsalva maneuver, stress tests and after pharmacological interventions, the substitution is not yet advised; however, studies are being conducted to validate this change [55,56].

### PPG Sensor

**Figure 5:**
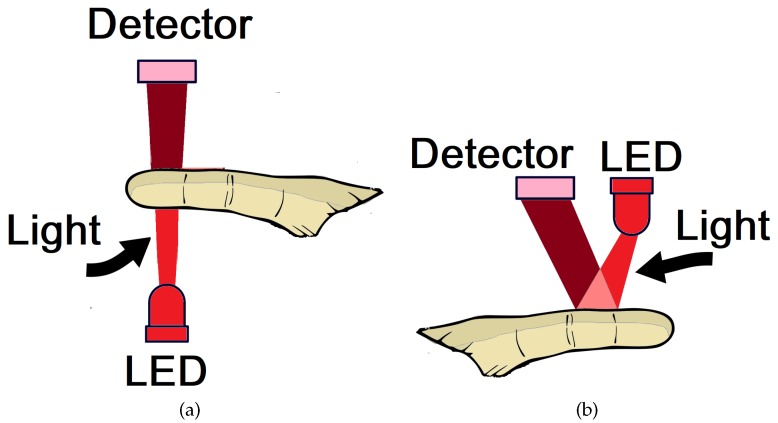
Representation of the operation of photoplethysmography sensors for finger application, by transmission (**a**) and by reflection (**b**). Adapted from [58].

Photoplethysmography sensors measure the amount of infrared light absorbed or reflected by blood. Volume changes are caused by pressure changes in blood vessels, which occur throughout the cardiac cycle [15]. There are two types of functioning principles for photoplethysmography sensors: the transmission or reflection of light through or by a certain part of the body [57]. The schematic representation of the PPG sensor is shown in Figure 5: the transmission operation (Figure 5a), in which the emission module and the photodetector are located on diametrically opposite sides; and by reflection (Figure 5b), in which the emission module is located on the same side as the photodetector.

With a PPG sensor in transmission mode, the LED light passes through absorbent substances, such as the skin pigmentation, bone and arterial and venous blood, and is then received by the detector and quantified by filters and converters [59,60].

In contrast, a PPG sensor in reflection mode reflects the LED light on the skin, which is received by the detector, and quantified in a similar fashion through the use of filters and converters. Nonetheless, this mode is applied mainly in the body parts too thick to allow the transmission of light (for example, wrist and forehead). Therefore, the PPG sensors can assume varied shapes, for example, a band, a wristwatch, or a patch. Additionally, some PPG sensors already make use of wearable technology, monitoring the heart rate in real time [59,60,61,62].

**Figure 6:**
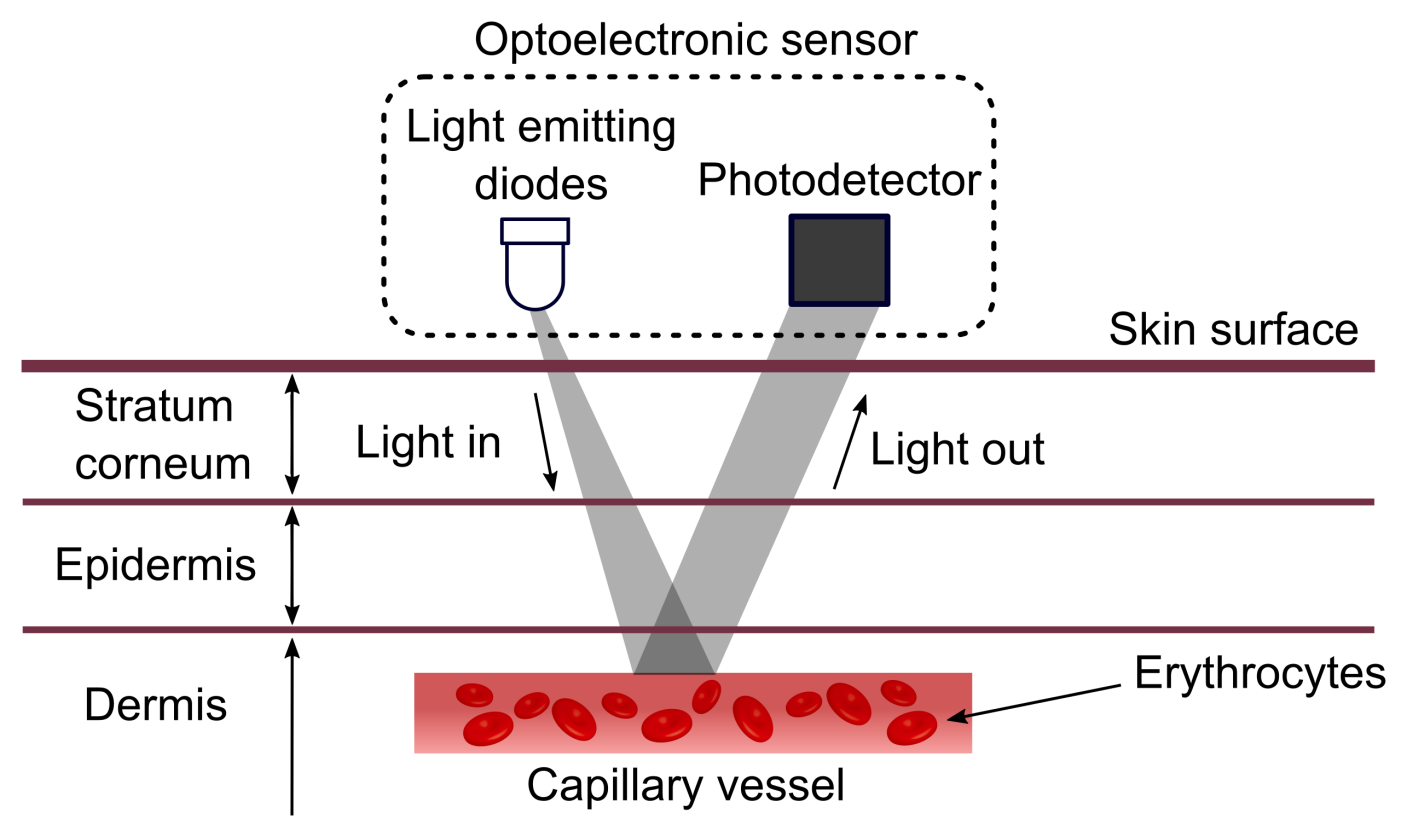
Working principle of PPG sensors [19].

The working principle of the PPG sensor is based on the emission of infrared light by an LED which penetrates the skin and blood vessels. This light is captured by the detector to measure the blood stream, as can be observed in Figure 6. The results of the PPG signal depend primarily on the flow of blood and oxygen to the capillary vessels in each heartbeat [19].

Theoretically, the PPG signal is formed by two components: (1) the DC offset, which represents the constant absorption of light passing through the tissues; and (2) the AC component generated by heartbeats affecting blood volume when light traverses the artery [19].

Regardless of the PPG sensor operating principle, it must be portable, lightweight, rugged, low cost and comfortable to use, besides keeping the signal quality under various conditions. There are several sites for measuring the PPG signal, such as the fingers and toes [63,64,65], forehead [66], wrist [67] and ear [62,68], since all of them have a rich arterial source and are relatively easy to attach a sensor.

PPG is based on the properties of light scattering caused by glucose in blood. The increase in glucose decreases the misalignment of the light beam penetrating the tissue, because the refractive index is reduced by its presence. As a result, a smaller amount of light is absorbed, and the light intensity which crosses the tissue is greater [69]. The PPG technique is based on Beer–Lambert’s law, which shows that light intensity decreases exponentially when traveling in an absorbent medium, and absorption is wavelength dependent [70].

Due to physiological particularities for each person, characteristics such as skin tone, thickness of the fat layer and rigidity of the radial artery have huge intervention in the morphology and amplitude of the plethysmographic wave. The Beer–Lambert law relates the intensity of the emitted to the incident light, in function of light absorption by the medium, the concentration of the solution, and the path the light travels. The higher is the luminosity emitted by the photo-emitter (LED), the higher is the amount of light transmitted through the medium as well as the amount of light reflected [69].

The preference for the PPG technique for heart and respiratory rate acquisition, rather than other techniques such as electrocardiogram (ECG), is due to the safer extraction of respiratory data, since the PPG waveform provides estimates better than those derived from ECG signal by means of respiratory sinus arrhythmia (RSA) analyses, as well as to the low cost of the PPG sensor [71]. The photoplethysmography technique may be used for both the prevention as the detection of various diseases. Therefore, the importance of the PPG approach for measurement and monitoring of the HRV is perceived.

## 3. Photoplethysmography Signal Processing and Analysis

For the analysis and understanding of the variables obtained through the measurement of HRV, some methods can be used, such as linear and nonlinear methods. Linear methods are divided into two types: time domain analysis, performed by using statistical and geometric indices, and frequency domain analysis [72]. The parameters extracted from the measurement of the PPG signal both in the time domain as the frequency domain can provide valuable information about the control of the cardiovascular system [73].

Obtaining HRV indices is of great importance for clinical understanding of certain physiological variables, since increases in variability indicate good physiological adaptation of the organism and its maintenance, thus predicting a condition of stability of the biological system, whereas reductions have been pointed out as predictors of diseases [72].

### 3.1. Time Domain—Statistical Indicators

The records for analysis of HRV indices using linear methods can be obtained in short periods (2, 5, or 15 min) or in long periods (24 h), which is more common in clinical practice [74]. For this analysis, at least 256 beat-to-beat intervals are recommended [75].

The statistical time-domain indices obtained by the beat-to-beat determination are: [76,77]

- SDNN—standard deviation of all $t_{1}$ intervals read in a time interval, expressed in ms;
- SDANN—standard deviation of the means of the intervals $t_{1}$, every 5 min, in a time interval, expressed in ms;
- SDNNi—the mean of the standard deviation of the intervals $t_{1}$ every 5 min, expressed in ms;
- rMSSD—the square root of the square mean of the differences between adjacent intervals $t_{1}$, in a time interval, expressed in ms; and
- pNN50—the percentage of the adjacent $t_{1}$ intervals with duration difference greater than 50 ms.

The SDNN, SDANN and SDNN${}_{i}$ indices are obtained from long-term records and represent sympathetic and parasympathetic activities, but they do not allow differentiation when HRV changes are due to increased sympathetic tone or withdrawal of vagal tone [74]. On the other hand, the rMSSD and pNN50 indices represent the parasympathetic activity, as they are found from the analysis of adjacent RR intervals [76,77].

### 3.2. Time Domain–Geometric Indices

Other methods used for HRV measurement and analysis are the geometric methods, which allow the presentation of cardiac pulse intervals (systole and diastole) and use approximations to derive the HRV measurements [78,79]. The main geometric methods used are:

- Triangular index (RRtri);
- Triangular interpolation of RR intervals (TINN); and
- Plot of Poincaré.

The triangular index and the TINN are calculated from a histogram of density of heart rate intervals (systole and diastole) which contains, on the X-axis, the length of the intervals of the pulses and, on the Y-axis, the frequency with which they occurred. Connecting the points of the columns of the histogram form a figure similar to a triangle, from which these indices are extracted [7,75,79].

The Poincaré plot is a two-dimensional graphical characterization of the correlation between consecutive cardiac pulses [7,78,80]. Some authors consider the Poincaré plot as based on nonlinear dynamics [81,82,83]. The Poincaré plot is a graphic representation of the correlation between intervals of consecutive heartbeats. A common manner describing the plot geometry is to fit an ellipse in the graph [82].

For quantitative analysis of the plot, by the adjustment of the ellipse of the figure formed by the attractor, the following indices are calculated: SD1 (standard deviation of the instantaneous variability beat-to-beat), SD2 (standard deviation of long term of the interval between heartbeats) and the ratio SD1/SD2 [81].

The index SD1 depicts the dispersion of points perpendicular to the identity line and is an index of instantaneous recordings of the variability beat-to-beat; SD2 describes the dispersion of the points along the identity line and represents the HRV in long duration recordings; and the relationship between both (SD1/SD2) represents the ratio between the short and long variations of intervals of the heart rate [7,84].

In individuals with COPD, a smaller dispersion of NN intervals is observed, both beat-to-beat and long-term, forming a characteristic image of HRV reduction. In healthy subjects, the intervals between the heartbeats are irregular, making it appear, in the Poincaré plot, as a cloud of points.

The qualitative (visual) analysis of the Poincaré plot is performed through the analysis of the figures drawn by the plot attractor. These can be classified as [82]:

1. Figure similar to a comet, in which an increase in the scattering of the beat-to-beat intervals is analyzed, characteristic of a normal plot;
2. Figure similar to a torpedo, with slight global beat-to-beat scattering (SD1) and without increasing the scattering of long-term beat-to-beat intervals; and
3. Parabolic or complex figure, in which two or more distinct ends are separated from the main body of the plot, with at least three points included in each end.

### 3.3. Frequency Domain

The linear method may also be applied in the frequency domain. In this perspective, the spectral power density method is the most frequently used method when dealing with individuals under resting conditions [75].

The analysis of spectral density evaluates how the power (variance) is distributed as a function of frequency. This analysis is done by using the properties of mathematical algorithms [75].

The frequency domain analysis is delimited in three distinct frequency bands, called spectral components, independently of the calculation of the spectral density (Fourier Transform Techniques or auto regressive model) [85,86]. These are:

- High frequency (HF) (0.15 to 0.40 Hz), modulated by the parasympathetic nervous system and generated by breathing;
- Low frequency (LF) (0.04 to 0.15 Hz); and
- Very low frequency (VLF) (0.01 to 0.04 Hz), modulated by both the sympathetic nervous system and the parasympathetic nervous system.

For analysis in the frequency domain, the spectral indices undergo some mathematical processing, forming a tachograph, which is a graph that expresses the variation of the beat-to-beat intervals as a function of time, such as Fourier transform (FFT) or autoregressive models (AR) [5]. The FFT method is used to obtain an approximation of the spectral power of the HRV. On the other hand, in the AR model, the estimation of the parameters can be easily done by solving linear equations [37].

The analysis of the power spectral density (PSD) is one of the most used approaches for investigating the autonomic control of the cardiovascular system. Two main components around 0.1 Hz (LF) and 0.25 Hz (HF) were studied. Through this analysis, it was possible to determine [56]:

(1) The HF component corresponds to the respiratory rhythm and is a vagal modulation marker.
(2) The LF component indicates the sympathetic activities.
(3) The reciprocal relationship between the two characterizes the simpato-vagal balance.

Another manner of analyzing the PPG signal in the frequency domain for verification of the thermal stress of an individual is the application of derivatives to the signal, the first derivative of the signal represents the blood speed and the second derivative represents the acceleration of the blood flow inside the tip of the finger, when this is the local of measuring the signal. Hence, it is expected that the application of derivatives amplify the differences between the PPG signals measured before and after the induction of thermal stress [87,88].

### 3.4. Nonlinear Methods

The heart rate measurement can also be studied by methods based on chaos theory, that is, by the theory of nonlinear systems [89,90]. The main nonlinear methods used to analyze HRV are: analysis of trend fluctuations, correlation function, exponent of Hurst, Fractal dimension and the exponent of Lyapunov [7,89]. Abnormal beats checked, for example by PPG, have inherent components of Brownian motion, while normal PPG is anti-persistent. The Hurst exponent is a dimensionless estimator of this trend of time series [91].

In the work [92], four different nonlinear methods were applied, Scaled Amplified Analysis (RSA), Higuchi Fractal Dimension (HFD), Displaced Flotation Analysis (DF A) and Exponential Generalized Hurst (GHE), to extract resources for authentication of the ECG signal and study the nonlinear properties of this signal. The proposed approach was tested using 18 ECG signals from individuals with normal sinus rhythm. The results show that the accuracy of the authentication is 99.06%.

A Lyapunov exponent is a real number that measures the average rate of divergence or convergence along the entire attractor that can be considered the point space or set of points representing several possible stationary states of a dynamic system. Therefore, this exponent can be used to study the stability of a system. The Lyapunov exponent may be positive (chaotic), zero (periodic), or negative (a fixed point). It is of greater interest to determine the largest exponent of Lyapunov (LLE) because it notion of predictability for a dynamic system [93].

In the work [93], the LLE was used to extract a useful characteristics of the PPG signals. The use of nonlinear methods was used in the work of [94] to analyze the behavior of PPG in subjects who presented fatigue, because for those individuals, PPG signal seemed a random signal. Although randomized systems may be random, they are actually deterministic systems governed by rules of complex or nonlinear materials, that is, one can find regularities or rules of phenomena that appear to be without regularities or predictability of the points of view from the chaos. In the cited work, the Lyapunov exponent was used, using dimensionality reduction, trying to relate the fatigue and the degree of chaos of PPG.

The general analysis of the main methods used for the analysis of the PPG signal, the domain, and the indices evaluated in each method are presented in Table 1.

## 4. Instrumentation

For the acquisition of the PPG signal and the information contained in the same, it is necessary to collect the signal from the body of the patient for prolonged periods. This is why it is important to condition the photoplethysmographic signal to instrumentation circuits to avoid the artifacts of movement as much as possible. The PPG signal presents low amplitudes, and therefore, noise heavily affects the quality and reading of the signal parameters, i.e., this signal is affected by various noises, such as the environment, the patient’s condition, breathing or movement. Each type of noise covers a range of frequencies [95,96]. For example, the respiratory rate range is 0.04–1.6 Hz, and the frequency range of the motion artifacts caused by patient movement is 0.1 Hz. The pulse wave frequency values of the PPG signal is in the range of 0.5–4.0 Hz. The frequencies of the motion artifacts and the PPG signal therefore overlap, making it impractical to separate them using classical filtering methods [96].

As shown in the previous sections, the PPG sensor consists simply of an infrared light emitting LED and a infrared light receiver phototransistor. Still, for the analysis of this signal it is also necessary to make use of filtering and amplification circuits. In [97], the signal obtained in the emitter of the phototransistor is filtered with a fourth-order low-pass filter with a cut-off frequency of 10 Hz to eliminate the high frequency noise. Furthermore, the DC tracking method was applied to eliminate the DC component of the signal. This method avoids the implementation of a high-pass filter with a very low cut-off frequency. Then, another fourth-order low-pass filter with a cutoff frequency of 0.7 Hz eliminates both the DC component and the artifacts. This signal is then subtracted from the original signal and amplified in an INA128 instrumentation amplifier with a gain of 10. Finally, the signal is applied to an operational amplifier of signal gain of 100. Subsequently, all this was reapplied to further clean the signal. After this analog processing, the signal can be used in a microcontrolled circuit.

In contrast, in [98], the circuit is composed simply of a combination of a set of LEDs driven by a MOSFET plus a photodetector for reading the signal. This is implemented by a single integrated circuit, the OSRAM SFH 7050 sensor. This component is a fully integrated optoelectronic sensor designed and optimized specifically for PPG signals. It exhibits three different light emitters plus a detector, also presenting a light barrier to minimize the optical crosstalk between the emitters and the detector, improving the signal-to-noise ratio [99]. The photodetector signal is applied in a transimpedance amplifier (TIA), which is a current to voltage converter implemented by an operational amplifier. Although a transimpedance amplifier is a good method to convert current to voltage, much care must be taken during the design of this kind of circuit, since it is prone to oscillate. The integrated circuit used as a transimpedance amplifier was the Microchip MCP6024, an operational rail-to-rail input and output amplifier. For this project, no filtering of the photoplethysmography signal was implemented on the final plate, since the signal is further processed by the software. However, low-pass and high-pass filters can be implemented to reduce computational cost.

**Figure 7:**
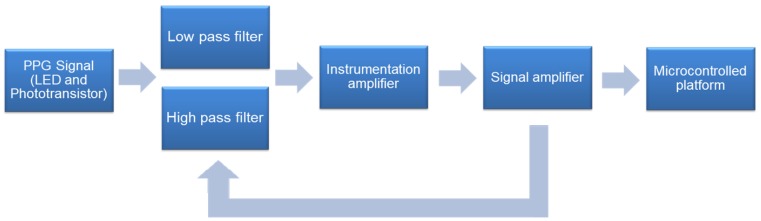
PPG instrumentation.

In the literature, many papers related to the development of PPG sensor instrumentation circuits can be found. When analyzing these works, it is verified that the instrumentation is based on the flowchart of Figure 7. Figure 7 presents a sequence of established steps for instrumentation of the PPG sensor, based on the work of [98,100,101,102,103].

## 5. Related Work and Clinical Applicability

HRV is used in investigations of cardiac autonomic function in areas such as Chagas disease, diabetes mellitus, heart failure, myocardial infarction, chronic obstructive pulmonary disease, etc. In addition, such technique is applied in the evaluation of athletes and non-athletes during physical training programs [1].

The normal range of the HR is between 50 bpm and 99 bpm. Nonetheless, in adverse conditions, this rate can increase or decrease, being indicative of an atypical situation [29].

Values above 99 bpm of heart rate denote a condition called tachycardia, indicating an increase in body temperature, stimulation by sympathetic nerves and heart toxicity. On the other hand, heart rate values below 50 bpm denote a state of bradycardia, a condition opposed to tachycardia, indicating vagal stimulation [104].

According to [1], HRV indices may describe functional and structural changes in the cardiovascular system due to the increase in the age of the human being, thus impacting the cardiac autonomic function. The self-monitoring of glycemia, an intermittent measurement of capillary glycemia, uses the glucometer as its main method. This technique is limited as for the number of measures that can be performed per day once it is invasive and painful. Some studies have related PPG as an effective method for measuring blood glucose levels by monitoring heart rate variability [104].

According to [105], the utilization of remote health monitoring systems presents itself as a very effective method in the assistance of patients with chronic conditions, such as the case of patients with heart diseases, which can be verified in the following examples.

A virtual instrumentation based tool was developed to assist the health professionals in the diagnostic of cardiovascular diseases, in which besides the recording of the blood pressure, the professional could verify other useful parameters, such as the history of the systolic, diastolic, and average pressure, in addition to the heart rate, adopting as the base of the measurement of HRV, photoplethysmography [11].

The authors in [70] developed a pulse oximeter device with the PPG technique to facilitate the diagnosis of childhood pneumonia in remote areas. In [1,106], the researchers used the time domain method to measure HRV with the objective of studying the characteristics of the cardiac autonomic behavior pattern of patients with chronic obstructive pulmonary disease. In [107], they carried out a study on the technique of photoplethysmography for monitoring HRV in the performance of plastic and reconstructive surgeries.

The authors in [29] developed a system for telemetric acquisition of physiological signals of patients in cardiovascular rehabilitation programs using PPG as the basis for HRV measurement. This system helps health professionals responsible for the cardiopulmonary rehabilitation session in the monitoring and evaluation of patients’ physiological parameters during physical exercise.

The HRV analysis has been widely used to understand the phenomena related to Autonomic nervous system under normal and pathological conditions. However, studies related to its use in clinical practice are still scarce. In [72], the authors have demonstrated the great potential of HRV analysis for clinical practice.

In Table 2, we show some studies relating the HRV measurement, either by the PPG or ECG technique, as an important prognostic factor for several cardiovascular diseases, as well as the evaluated indices and the respective results of these studies.

Several studies relate different technologies for the analysis of the PPG signal. Such studies essentially vary as for the sensor used and for the methods of monitoring HRV through PPG. Some of these studies are briefly presented next. In [117], the authors produced a prototype, at both software and hardware levels, with the objective of assisting the stress diagnosis, by using photoplethysmography as an HRV measurement technique.

The researchers in [71] designed a device that includes an integrated hardware and software solution for obtaining heart rate in beef cattle, since cardiac information is essential for analyzing animal health. The authors of [118] developed a prototype based on the PIC18F4550 microcontroller for monitoring the oxygen saturation and heart rate of rodents, by applying photoplethysmography as a technique for measuring HRV.

In [64], the authors designed a monitoring and training system for cyclists with smartphones, using photoplethysmography to measure the cyclist’s physiological signals such as heart rate and oxygen percentage. By integrating the GPS receiver of the smartphone and the developed software, it was possible to take the measures of distance and speed covered by the rider.

In Table 3, we present a summary of the work regarding heart rate variability with ECG and PPG techniques with different technology proposals. The measurement of HRV by applying the PPG technique has been studied as for the properties of the extracted signal. This signal can be detected in many activities and responses such as heart rate, breathing rate, oxygen saturation, blood viscosity, blood pressure or changes in the user’s position. Thus, choosing the suitable equipment and technique of extraction of signal details is of fundamental importance for studying the PPG signal [117].

## 6. Discussion and Open Issues

While studying the works selected for this review, we noticed that several of the proposed techniques presented potential for the construction of medical diagnostic assistance tools. We also noticed that several studies have correlated the PPG with the ECG to HRV measurement.

The measurements of SpO${}_{2}$ and HR by the non-invasive PPG technique are being largely employed in personal portable devices and clinical pulse oximetry due to its convenience and capacity of to perform continuous readings [13].

With the progress in the study of the PPG technique and the analysis of the devices most commonly used, the necessity of miniaturization of the measurement device or improvement in the technology of the equipment was perceived. These advancements in the non-invasive physiological detection, miniaturization of the hardware, and wireless communication are leading to the development of new wearable technologies having wide and important implications to the health area [67]. Wearable computation has the potential of revolutionizing health care implementing low-cost physiological monitoring, in addition to enabling comfortable a continuous cardiovascular monitoring away from the clinical ambient and during long periods of time [21,120].

In [63], a miniaturized sensor for continuous long-term monitoring, named “ring sensor”, was developed. It is attached to the base of a finger for beat-to-beat monitoring, while the data are sent to a host computer through a radio-frequency transmitter. Additionally, the PPG technique has its successful application in smart watch technology, a smart watch which, besides the basic functions of a watch, provides some functions common to smartphones. One of these functions is the monitoring of cardiac activity by PPG; the smartwatch reports in its screen the values of the individual’s heart rate. However, it has the downside that only the HR value is presented on its screen, not the the waveform of the PPG signal, being the last quite employed in the detection of cardiac anomalies.

The rapid growing of Internet of Things (IoT) technology and biosensors resulted in new opportunities for personalized services of e-health and health, one of these services is the interconnection between the PPG technique and the Internet of Things technology [21,120,121,122]. The popularity of portable sensors and the Internet of Things (IoT) transfer significant privileges to the body sensors networks which could communicate which the cloud computing platforms to offer the interoperability in the monitoring of health and welfare [123,124].

With the interconnection of these two technologies arose the concept of Internet of Health Things (IoHT) which is essentially one IoT based solution comprehending a network architecture that allows the connection between a patient and health installations, for example, e-Health systems based in IoT for electrocardiography, heart rate, electroencephalogram, diabetes and other different types of body (vital) signs monitoring based in biomedical sensors [125]. These are capable of monitoring the pulse signal, blood oxygen (SPO${}_{2}$), air flow, body temperature, arterial pressure, patient’s orientation and electromyography. These data are processed by applications developed for a user terminal, such as computers, smart phones, smartwatches or even specialized devices [126,127,128].

As an example, the work of Constant et al. [129], who developed a device called Pulse-Glasses, is connected to the cloud and and able to monitor the heart rate with the PPG technique. The IoT functionalities were implemented in a manner where the HR data are registered from the Pulse-Glasses, visualized in a Android smartphone Android and saved in the cloud. Consequently, the monitoring results can easily be sent to a doctor or a hospital database.

In [130], photoplethysmography signals were harvested and utilized to calculate the heart rate and oxygen saturation. The system developed is capable of providing feedback to the user through a smartphone application, which receives the PPG signals from the device through Bluetooth communication, while being able to send a notification with the exam results to the user’s doctor.

One of the problems encountered in the utilization of the photoplethysmography technique is that the conventional contact PPG sensors are not suitable for situations where the skin has been damaged or when it is necessary to allow movements without restrictions. Besides, it can be noted that the pressure of the conventional finger-clip sensors can alter the waveform of the PPG signal due to the contact force between the finger and the sensor. Furthermore, the PPG signals are highly susceptible to movement, which makes their use in cardiopulmonary exercise testing or cardiopulmonary resuscitation difficult [131,132]. There are already research works demonstrating that this problem can minimized or even solved, as it is the case in the work of Yuan et al. [133], who produced a method of improvement for minimization of the artifacts due to the movement performed with a PPG sensor.

The effects occasioned by noise and artifacts to the PPG signal can be reduced in different ways through the adequate processing of the PPG signal. More basic filters can aid in the reduction of artifacts, e.g. the moving average filter, which is highly used in this application, works well for a limited range of artifacts [134].

Another method is the application of adaptive filters, which cope effectively with the band noise, needing a signal reference. In most cases, adequate reference signals were obtained making use of additional hardware. For example, the reference signals were obtained from an additional transducer connected to identify the movement or employing a additional type of optoelectronic reflectance sensor [135,136].

Due to the dynamic nature of the biological systems, most of the biological signals are non-stationary and alter substantially their properties over time [134]. Time–frequency methods such as the wavelet transform and the smoothed pseudo Wigner–Ville distribution can be applied to PPG signals showing significant improvement in comparison to traditional approaches [134,137].

Although the artifact reduction approach in the PPG signal by the Wavelet transform has been examined in the last decades, it is still considered an excellent method for the reduction of motion artifacts in the PPG signal. Because PPG contains information related to heart rate, heart rate variability, blood pressure, and respiration, Wavelet can be efficiently identified to preserve respiratory induced intensity variation while removing PPG signal artifact movements [137,138,139]. Because motion artifacts result in in-band noise, adaptive filters offer the best solution compared to conventional ones such as the moving average filter [135,136,140].

In [141], a study was carried out to investigate the effects of the electromagnetic field on extremely low frequency in response to photoplethysmographic (PPG), electrocardiographic (ECG) and electroencephalographic (EEG) activity. With this, the wavelet transform was analyzed as a characteristic extraction method to represent the electrophysiological signals.

The Wavelet transform and its possible derivations are increasingly gaining space as a method of artifact reduction in the PPG signal, as in the work in [142], which proposes an approach to reduce photoplethysmographic signal movement artifacts (PPG) based on the concept of a double tree complex wavelet transform technique. The processing of corrupted PPG motion artifacts with this technique has outperformed db10 wavelet processing and can be referred to as the best technique for reducing movement artifacts suitable for pulse oximetry applications.

The PPG technique has its advantages and disadvantages, however the benefits of this technique applied with diverse technologies can culminate in the advance in the fields of both medicine and biomedical engineering, aiding in the early detection of a cardiopathy or improving the medical supervision of a cardiac patient.

## 7. Learned Lessons

In this work, it was perceived that the heart rate variability is one important tool for the analysis of the physiological conditions of a patient, as well as an auxiliary method for the diagnosis of cardiopathies. The PPG technique is a simple method of monitoring the HRV and frequently better known for its use in pulse oximeters. Research regarding this technique has been showing significant progress, such that some researchers in the medical and biomedical engineering fields are giving preference to utilization of this technique over ECG.

The benefits of the PPG approach compared to ECG are greater than the drawbacks, bearing in mind that it is a simpler technique and there is not the necessity of attaching electrodes to the patient’s chest. The expansion of the application of PPG together with the advance of the industry of health care is notorious. This type of interconnection can support many medical conditions, including medical care of pediatric patients and the eldery, chronic diseases management of private health care and fitness, among others. Thus, it is perceived that this type of industry is between the fastest in accepting IoT based solutions, and it is fact that IoHT will create a significant economical impact in the world.

The extensive utilization of this technique is related to its simplicity, convenience, easiness of connection with IoT technologies, and for it being a non-invasive approach. The association of this technique with the advance of technology tend to proportionate the diagnosis of cardiopathies precociously, improve the treatment of the patients with the management and monitoring of the patient remotely in a healthcare ambient and lower the number of deaths caused by cardiovascular diseases.

## 8. Conclusions

After a review of the literature published in the last 20 years related to studies of photoplethysmography, a significant expansion in the utilization of this technique as a method of measurement and monitoring of the HRV was observed.

A large proportion of these studies published, besides mentioning PPG as an approach for measurement and monitoring HRV, present this technique as effective for the diagnosis of several cardiovascular diseases. This is only possible when interlinking the technique with IoT technologies, genetic algorithms and artificial intelligence. For this reason, a closer relationship between the researchers of the biomedical engineering field and the medical community is necessary to provide a deeper investigation of this topic, facilitating new studies which can help doctors in the early diagnosis of some cardiac diseases.

Thus, this article can aid researchers, developers and professionals in the clinical field in the study of the heart rate variability and the PPG as a tool in the diagnosis of chronic diseases. It can also be a source of information for healthcare providers and specialists interested in IoHT.

## Figures and Tables

**Table 1:** Summary of the methods used for the analysis of PPG signal.

Method	Domain	Evaluated Indices
Linear	Time domain	Statistical indices: SDNN, SDANN,
		SDNN${}_{i}$, rMSSD, pNN50.
Linear	Time domain	Geometric indices: RRtri, TINN and
		plot of Poincaré.
Linear	Frequency domain	HF, LF and VLF.
Nonlinear	-	Correlation function, hurst exponent,
		fractal dimension and the
		*Lyapunovexponent*.

**Table 2:** Clinical applicability of Heart Rate Variability.

Reference	Year	Disease	Evaluated Indices	Results
[70]	2013	Pneumonia	HRV and PPG signal	Mean squared error of 3.0 breathing/minute.
[108]	2005	Peripheral arterial occlusive disease of the lower limbs (PAOD)	PPG signal	90% of accuracy and 100% of sensitivity.
[109]	2011	Obesity	HF, LF and VLF	Low levels of excess fat in eutrophic young increase cardiovascular risk.
[110]	2012	Respiratory sleeping disorders in patients with severe cardiovascular disease	PPG, EEG, ECG and EMG	Sensitivity of 98%, and specificity of 96%.
[111]	2011	Chronic heart failure (CHF)	Frequency and time domain	89.74% sensitivity and 100% of specificity.
[112]	2002	Coronary heart disease	SDNN, HF	HRV can be used for identifying differences in the cardiac autonomic balance of healthy adults.
[113]	2015	Childhood pneumonia	Respiratory rate, HRV and SPO2	96.6% of sensitivity, 96.4% of specificity.
[106]	2007	Chronic obstructive pulmonary disease (COPD)	SDNN, RMSSD, HF, LF	Reduced HRV with decreased sympathetic and vagal activity.
[114]	2011	Respiratory sinus arrhythmia	HF	Mean error in RR detection of 0.05 to 4.23 breathing/minute for PPG and 1.59 to 3.70 breathing/minute for ECG.
[115]	2008	Renal insufficiency	SDNN, LF	Chronic renal patients not undergoing dialysis have reduced HRV.
[116]	2015	Cardiovascular risk (CR)	Pulse, SpO_2_ and PPG signal	Technical error of 0.8% and 1.0%.
[2]	2011	Peripheral arterial occlusive disease (PAOD)	Time domain	The PPG signal amplitude and distortion increases with disease severity.

**Table 3:** Technology proposals for measurement and monitoring of HRV.

Reference	Year	Technique	Proposal
[105]	2012	ECG	Application to assist in remote
			monitoring of cardiac patients.
[117]	2010	ECG e PPG	Device for measuring the level
			of stress of an individual.
[11]	2006	PPG	Low-cost prototype for blood
			pressure measurement.
[119]	2012	PPG	A new prototype fiber–optic
			probe was developed for
			investigating PPG signals
			from various splanchnic organs.
[25]	2016	PPG	Measurement of HRV through
			hand image.
[64]	2012	PPG	Wireless system for monitoring
			and training cyclists.
[70]	2013	PPG	Portable oximeter to aid in the
			diagnosis of childhood pneumonia.
[27]	2016	PPG	Measurement of HRV by
			facial detection.
[71]	2014	PPG	Obtainment of HRV in beef cattle.
[118]	2008	PPG	Oxygen saturation and heart rate
			monitoring system for rodents.
